# Acetate Metabolism and the Inhibition of Bacterial Growth by Acetate

**DOI:** 10.1128/JB.00147-19

**Published:** 2019-06-10

**Authors:** Stéphane Pinhal, Delphine Ropers, Johannes Geiselmann, Hidde de Jong

**Affiliations:** a Univ. Grenoble Alpes, CNRS, Laboratoire Interdisciplinaire de Physique, Grenoble, France; b Univ. Grenoble Alpes, Inria, Grenoble, France; University of Illinois at Urbana Champaign

**Keywords:** *Escherichia coli*, acetate, acetate metabolism, growth inhibition, metabolic flux analysis, overflow metabolism

## Abstract

During aerobic growth on glucose, Escherichia coli excretes acetate, a mechanism called “overflow metabolism.” At high concentrations, the secreted acetate inhibits growth. Several mechanisms have been proposed for explaining this phenomenon, but a thorough analysis is hampered by the diversity of experimental conditions and strains used in these studies. Here, we describe the construction of a set of isogenic strains that remove different parts of the metabolic network involved in acetate metabolism. Analysis of these strains reveals that (i) high concentrations of acetate in the medium inhibit growth without significantly perturbing central metabolism; (ii) growth inhibition persists even when acetate assimilation is completely blocked; and (iii) regulatory interactions mediated by acetyl-phosphate play a small but significant role in growth inhibition by acetate. The major contribution to growth inhibition by acetate may originate in systemic effects like the uncoupling effect of organic acids or the perturbation of the anion composition of the cell, as previously proposed. Our data suggest, however, that under the conditions considered here, the uncoupling effect plays only a limited role.

**IMPORTANCE** High concentrations of organic acids such as acetate inhibit growth of Escherichia coli and other bacteria. This phenomenon is of interest for understanding bacterial physiology but is also of practical relevance. Growth inhibition by organic acids underlies food preservation and causes problems during high-density fermentation in biotechnology. What causes this phenomenon? Classical explanations invoke the uncoupling effect of acetate and the establishment of an anion imbalance. Here, we propose and investigate an alternative hypothesis: the perturbation of acetate metabolism due to the inflow of excess acetate. We find that this perturbation accounts for 20% of the growth-inhibitory effect through a modification of the acetyl phosphate concentration. Moreover, we argue that our observations are not expected based on uncoupling alone.

## Abstract (precis)

High concentrations of organic acids such as acetate inhibit growth of Escherichia coli and other bacteria. This phenomenon is of interest for understanding bacterial physiology but is also of practical relevance. Growth inhibition by organic acids underlies food preservation and causes problems during high-density fermentation in biotechnology. What causes this phenomenon? Classical explanations invoke the uncoupling effect of acetate and the establishment of an anion imbalance. Here, we propose and investigate an alternative hypothesis: the perturbation of acetate metabolism due to the inflow of excess acetate. We find that this perturbation accounts for 20% of the growth-inhibitory effect through a modification of the acetyl phosphate concentration. Moreover, we argue that our observations are not expected based on uncoupling alone.

## INTRODUCTION

Growth rate is probably the most important physiological parameter characterizing bacteria. The growth rate of a bacterial culture depends on the composition of the growth medium and the genotype of the particular strain. Under the most commonly used controlled growth condition, minimal medium supplemented with glucose as the sole carbon source, the model bacterium Escherichia coli secretes acetate, a by-product of glycolysis, during fast aerobic growth. This “overflow metabolism” is a function of growth rate. Experiments that vary the rate of glucose utilization by E. coli cells growing aerobically show a linear increase of growth rate, with the rate of glucose utilization up to around 0.6 h^−1^ (1). Beyond this growth rate, respiration becomes limiting at 15 mmol of O_2_ per g of dry weight (gDW) and per h. Since glucose can no longer be fully oxidized to CO_2_, the extra redox potential is eliminated by secreting metabolites such as acetate (2). These observations have been explained in terms of constraints on proteome allocation. Above a certain glucose uptake rate, the cell favors the use of fermentation pathways that are less efficient than respiration in producing ATP but are also less costly to synthesize (3).

The secretion of acetate and other fermentation acids during growth is common in microorganisms, and it has been known for a long time that acid accumulation in the medium inhibits growth. For example, the growth rate of E. coli in minimal medium with glucose is reduced with increasing concentrations of acetate, diminishing to half of its reference growth rate in glucose alone when about 100 mM acetate is added to the medium (4). This inhibitory effect of acetate and other organic acids on microbial growth is of considerable practical interest. The addition of organic acids is widely used in the food industry to inhibit the growth of microbial pathogens (5). Moreover, growth inhibition by acetate and other organic acids is an important problem in biotechnological fermentation processes, limiting their utilization as a substrate for biorefining applications (6) and reducing the production of recombinant proteins in aerobic high-cell-density cultures (7, 8). This has motivated many studies in E. coli, searching for genetic modifications capable of reducing the flux to unwanted anaerobic by-products or increasing the acid tolerance of the cell (9–11; see references 7 and 12 for reviews).

Several hypotheses have been advanced in the literature to explain the inhibition of microbial growth by acetate and other organic acids. The classical explanation invokes the uncoupling effect of organic acids. Acetic acid (HAc), the protonated form of acetate, can diffuse freely across the cell membrane (13). Inside the cell, HAc dissociates into an acetate anion (Ac^−^) and a proton (H^+^) because the pK_a_ of HAc (4.76) is much lower than the intracellular pH (around 7.6 [14]). In order to maintain the membrane potential, the excess protons have to be expelled from the cell, which causes an energy expenditure detrimental to growth (15–17). The presence of acetate anions inside the cell also increases the internal osmotic pressure, which forms the basis for a second explanation (18, 19). Roe et al. have observed that, in order to maintain osmotic pressure, the intracellular pools of other anions, most prominently glutamate, are reduced (18). The resulting perturbation of anion pools may affect the functioning of metabolism and, therefore, growth. A follow-up study showed that high concentrations of acetate in the cell specifically inhibit a step in the biosynthesis of methionine, leading to the accumulation of the toxic intermediate homocysteine (20). The authors observed that growth inhibition could be substantially relieved by supplying the medium with methionine.

A recent study showed that, surprisingly, acetate is also taken up and consumed by E. coli cells growing on excess glucose (21). Further work confirmed that the PtA-AckA pathway not only produced but also consumed acetate (22). The net flux through the pathway was found to be controlled thermodynamically, in the sense that at high concentrations of external acetate, the flux direction is reversed and E. coli cells consume acetate while growing on glucose. This suggests a third hypothesis for growth inhibition by acetate, namely, the perturbation of acetate metabolism. The influx of excess acetate into the cell may be detrimental to maximum growth on glucose by perturbing fluxes in central metabolism. Moreover, it may change the concentration of acetyl-phosphate (Ac∼P), a signaling metabolite that can transfer phosphate groups to regulatory proteins and thereby modulate the expression of many genes or affect other processes, such as motility (as reviewed in references 23 and 24). *In vitro* studies have suggested that Ac∼P even functions as an alternative phosphate donor in the uptake of sugars transported by a phosphotransferase system (PTS) (25). Moreover, Ac∼P is involved in the acetylation of enzymes and regulatory proteins with broad physiological consequences (26–28).

**FIG 1:**
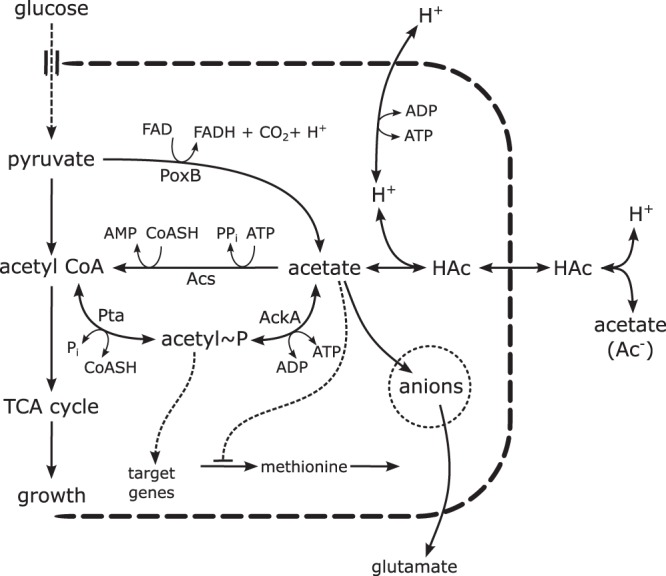
Schematic representation of the major metabolic pathways of acetate metabolism. Acetate can be generated directly from pyruvate by decarboxylation (using the enzyme pyruvate oxidase, PoxB) or from acetyl-CoA via the intermediate acetyl-phosphate (Ac∼P) (reactions catalyzed by the enzymes phosphotransacetylase, Pta, and acetate kinase, AckA). Acetate can freely diffuse across the cell membrane in the protonated form (13), HAc, or as an acetate ion, Ac^−^, via the transporter ActP or SatP (38). Protons that enter the cell in the form of HAc can be expelled at the expense of energy. The enzyme acetyl-CoA synthetase, Acs, efficiently converts intracellular acetate into acetyl-CoA. Acetate is also involved in several metabolic pathways. For example, the biosynthesis of methionine is inhibited by acetate. Excess intracellular acetate perturbs the anion balance in the cell and thus could inhibit other metabolic reactions (20). The pool of the major intracellular anion, glutamate, is strongly reduced when the intracellular concentration of acetate is high.

The major aim of this study was to investigate this third hypothesis, namely, that growth inhibition by acetate is connected with the influx of excess acetate into central carbon metabolism and/or with the regulation of cellular functions by Ac∼P. To this end, we constructed a collection of mutant strains with deletions of genes encoding enzymes involved in acetate metabolism. The metabolic network of acetate excretion and assimilation is represented in Fig. 1. We constructed mutants in all relevant genes coding for enzymes that connect acetate to central carbon metabolism and/or produce Ac∼P, i.e., the genes *acs*, *pta*, *ackA*, and *poxB*. We reasoned that if growth inhibition occurs through the uptake and assimilation of acetate by central carbon metabolism, with the consequent perturbation of fluxes and Ac∼P levels, this effect should be strongly mitigated in the mutant strains. Moreover, one would expect the distribution of fluxes within central carbon metabolism to be strongly perturbed by the addition of acetate to the growth medium. We tested these predictions by studying the effect of acetate on the growth of E. coli strain BW25113 under well-controlled growth conditions (minimal medium with glucose at pH 7.4 or pH 6.4) using the above-mentioned defined mutants of otherwise isogenic strains. Moreover, we quantified the extracellular concentrations of the sole carbon source under our conditions, glucose, and the major fermentation products: acetate, formate, pyruvate, lactate, and ethanol.

First, we found that in mutant strains devoid of Ac∼P (Δ*pta ackA* [Δ*pta ackA* is the shorthand notation for the Δ*pta* Δ*ackA* double mutant; an analogous abbreviation is used for the other strain descriptions]), growth inhibition is reduced by 20%. This indicates that Ac∼P has a small but significant effect in mediating the inhibitory effect of acetate. The same effect was found in the single Δ*ackA* mutant, which suggests that blocking the synthesis of Ac∼P from external acetate via AckA is enough to reduce the Ac∼P concentration to a level below which it no longer contributes to growth inhibition. Second, we computed uptake and secretion rates from the measurements of extracellular metabolite concentrations, both in the wild-type strain and in the Δ*acs pta* and Δ*acs pta ackA* strains. When combining the measured uptake and secretion rates with a genome-scale flux balance model (29), the predicted internal metabolic fluxes during growth with or without acetate are found to be strongly correlated. This suggests that, apart from a proportional rescaling of fluxes due to the reduced growth rate, central carbon metabolism functions in much the same way whether acetate is added to the medium or not. We conclude that the growth-inhibitory effect of acetate is not due to the influx of excess acetate into central carbon metabolism.

Our results indicate that changes in the concentration of Ac∼P account for about 20% of the reduction in growth rate in the presence of high acetate concentrations in the medium. Although the data do not allow us to unambiguously attribute the remaining 80% of the effect to either or both of the two classical hypotheses, uncoupling and anion imbalance, they do provide circumstantial evidence that the uncoupling hypothesis is less important than is sometimes assumed, consistent with previous reports (19, 30). In particular, we find that the biomass yield, defined as the ratio of the growth rate to the glucose uptake rate, does not significantly change when acetate is added to the medium, contrary to what is expected from the uncoupling hypothesis. Moreover, deletion of known acetate transporters does not noticeably change the growth-inhibitory effect of acetate, whereas it would be expected to affect the futile cycle of acetate uptake and secretion necessary for uncoupling. These observations, while not conclusive in themselves, provide an interesting basis for further research, in particular the measurements of the changes in bioenergetic parameters upon acetate addition and the precise characterization of physiological changes accompanying the perturbation of anion pools and their regulatory effects at the molecular level.

## RESULTS

### Growth inhibition by acetate.

In order to dissect the mechanism of growth inhibition by acetate, we adapted a standardized, well-controlled experimental setup from reference 4. We use the Escherichia coli BW25113 strain. E. coli bacteria from an overnight preculture in minimal medium supplemented with glucose were diluted into the same medium and grown in a shake flask batch culture. The growth characteristics of our strain are identical to those measured for similar strains of E. coli (31). As shown in Fig. S1 in the supplemental material, after about 7 h of growth, the bacteria reach a final optical density at 600 nm (OD_600_) of 3.5. pH and oxygen pressure decrease continuously during the exponential growth phase due to the increasing number of bacteria consuming oxygen and secreting acidic by-products. During the entire growth of the culture, the partial oxygen pressure, pO_2_, never falls below 40%, meaning that the bacteria grow aerobically (31). The pH remains close to neutral (pH 7) at the beginning of the experiment but drops to a value of 6.4 at the end of exponential phase (Fig. S1).

**FIG 2:**
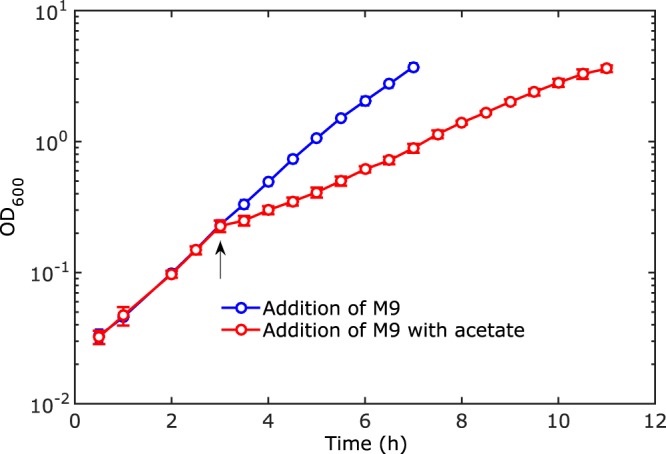
Growth inhibition by acetate. The inoculated culture is split and cultured in separate shake flasks containing minimal medium with glucose at pH 7.4. A small volume of minimal medium with acetate is added to one culture, and an identical volume of minimal medium without acetate is added to the second, control culture. The moment of acetate addition is indicated by the arrow. The optical density measurements represent the means from three experiments. The error bars (mostly smaller than, and therefore hidden by, the circles showing the data points) are two times the standard errors of the means. The data shown correspond to the wild-type strain and a final concentration of acetate of 128 mM.

In order to quantify the effect of acetate on the growth of the culture, identical starting cultures were split at the beginning of the experiment and grown in separate shake flasks. After about 3 h of growth, a solution with a defined concentration of acetate was added to the medium in one flask and a solution without acetate to the medium in another flask (Fig. 2). Growth of both the acetate-treated and the control culture were monitored until the end of exponential phase, and the OD_600_ and extracellular metabolites were measured at regular time intervals (see below). From these measurements we computed the growth rate, as described in Materials and Methods. The growth rate was determined from optical densities below 1, since above this value the oxygen pressure decreases to a point that it no longer allows growth at the maximum rate supported by the medium. Moreover, in the fast-growing control culture, acidification of the medium due to overflow metabolism sets in at higher optical densities (Fig. S1), confounding the effect from external acetate addition.

Growth inhibition by acetate is quantified using a so-called inhibition index (*i*) (4), defined as

(1) $$i=\frac{\mu_{\text{control}}-\mu_{\text{acetate}}}{\mu_{\text{control}}}$$

where μ_control_ denotes the growth rate in the culture without added acetate and μ_acetate_ the growth rate in the culture with added acetate. Note that the value of the inhibition index varies between 0 and 1. An inhibition index of 0 means no growth inhibition by acetate, whereas an inhibition index of 1 corresponds to full growth inhibition.

**FIG 3:**
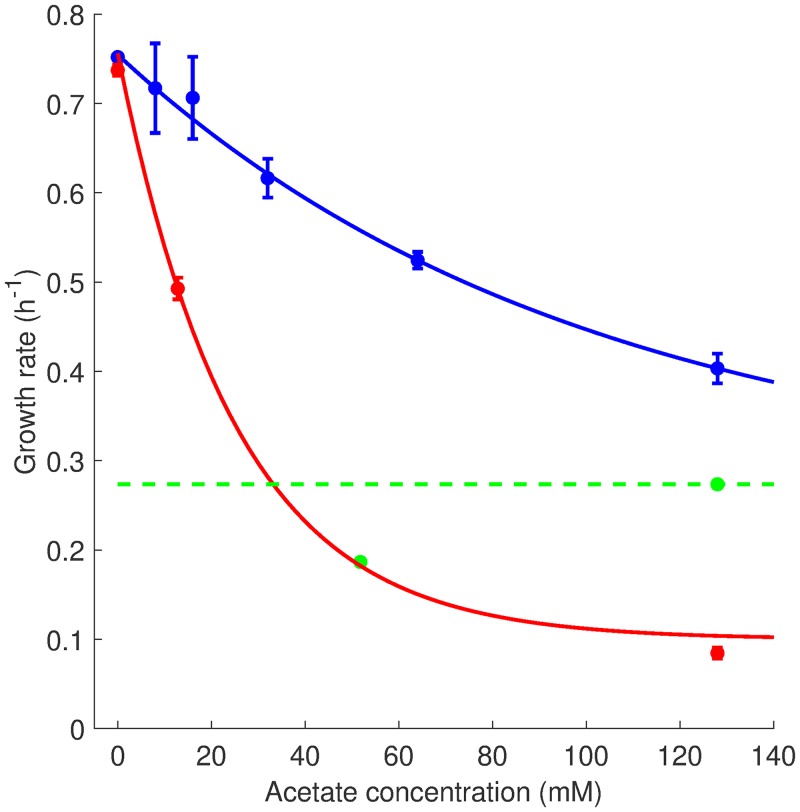
Growth inhibition by different concentrations of acetate and different pH levels. Experiments like those shown in Fig. 2 were carried out for different concentrations of acetate at pH 7.4 (blue dots) and pH 6.4 (red dots). Each data point shows the mean growth rate from three or four independent experiments, as well as an error bar equal to twice the standard errors of the means. An exponential function with baseline of 0.3 h^−1^ was fit to the data (blue and red curves). The baseline approaches the growth rate in minimal medium with 128 mM acetate as the sole carbon source (green line). For reference, the growth rate on 3 g liter^−1^ of acetate, corresponding to 50 mM, is also shown (green dot). Notice that for some measurements the error bar is so small that it coincides with the measurement dot.

Figure 3 shows the measured growth rate of the wild-type strain after the addition of different concentrations of acetate to a culture growing under the reference conditions at pH 7.4, close to the intracellular pH value enabling maximal growth (14). As can be seen, the growth rate drops from 0.75 h^−1^ to 0.4 h^−1^ when the acetate concentration increases from 0 to 128 mM. This change in growth rate corresponds to an inhibition index of 0.47. A previous report (4) had suggested an exponential decrease of growth rate as a function of the concentration of added acetate. When allowing for an offset of the exponential function, we obtain the best exponential fit with a baseline at 0.3 h^−1^. This baseline value corresponds to the growth rate of E. coli in the same medium with 128 mM acetate as the sole carbon source (0.27 h^−1^) (Fig. 3).

Given that growth inhibition is a monotonic function of the acetate concentration and that industrially relevant concentrations of acetate are on the order of 100 mM (32), we decided to carry out all subsequent experiments at 128 mM acetate. The qualitative effect of acetate is probably the same at all concentrations, but quantitative estimates are much more easily obtained for larger effects. While most measurements were carried out at pH 7.4, we also quantified growth inhibition by acetate at pH 6.4 and found an even stronger effect of acetate: the growth rate drops from 0.74 h^−1^ to 0.084 h^−1^ when adding 128 mM acetate, corresponding to an inhibition index of 0.89 (Fig. 3).

Previous results have shown that the addition of methionine to the medium can alleviate the inhibitory effect of acetate (18). Further work indicated that acetate inhibits methionine biosynthesis and, more particularly, the activity of the MetE enzyme (20, 33). As a further control, we therefore tested whether supplementing the growth medium with methionine could offset the inhibition effect. We added 128 mM acetate during growth in minimal medium with glucose at pH 7.4, in the absence of methionine and in the presence of 3.3 mM methionine (comparable to the concentration of 2 mM used in reference 18). We found only a small, nonsignificant effect of methionine under our conditions, consisting of an increase of the growth rate from 0.38 h^−1^ to 0.43 h^−1^ and a corresponding decrease of the inhibition index from 0.52 to 0.45 (Fig. S2). Roe et al. observed a much stronger effect, where supplementing methionine could restore 75 to 80% of the growth rate diminution due to acetate (18). More recent data also demonstrate a relief of growth inhibition by methionine, but the magnitude is closer to what we observed (reduction of inhibition index from 0.71 to 0.53) (33). Note that these experiments were carried out at pH 6, which may account for the difference in strength of the relief of growth inhibition by methionine observed under our conditions. For our purpose, however, the most important conclusion is that methionine is not a major contributor to growth inhibition at physiological pH levels. Therefore, in the remainder of the paper, we will investigate another hypothesis.

### Mutants of acetate metabolism.

While growing at a high rate under aerobic conditions, E. coli redirects some of the glycolytic flux toward the production of acetate, since limited respiration capabilities do not allow all carbon intermediates to enter the tricarboxylic acid (TCA) cycle (2). This physiological response optimizes the balance between demands for energy production and biomass synthesis (3). Conversion of acetyl coenzyme A (acetyl-CoA) into acetate involves the phosphotransacetylase Pta and the acetate kinase AckA. Acetate can also be produced from pyruvate, the metabolite just before acetyl-CoA in glycolysis, by pyruvate oxidase, PoxB (Fig. 1). When E. coli cells are growing on acetate, the Pta-AckA pathway operates in the reverse direction, converting acetate into acetyl-coenzyme A (AcCoA). For low concentrations of acetate in the medium, on the order of a few millimolars, the main route for acetate assimilation involves the acetyl-CoA synthetase Acs (23).

Recent work has shown that, during growth of E. coli on glucose, acetate metabolism not only produces but also consumes acetate, and acetate uptake or secretion corresponds to the net flux through the Pta-AckA pathway (22; see also reference 21). At high external concentrations, the net flux of acetate was observed to change direction and flow into the cells. This suggests that excess acetate flows into central metabolism and possibly perturbs fluxes necessary for sustaining maximal growth. Moreover, excess acetate may change the concentration of acetyl-phosphate (Ac∼P), the intermediate of the Pta-AckA pathway. Ac∼P plays an important role in the regulation of various cellular processes, as mentioned in the Introduction (see references 23 and 24 for reviews).

Valgepea et al. found that in continuous culture experiments at low dilution rates, even in the absence of acetate secretion, the Pta-AckA and Acs pathways are actively recycling acetate (34), possibly to maintain an appropriate level of Ac∼P. Under our conditions, Acs is not significantly expressed at the reduced growth rate following acetate addition (Fig. S3). However, the more general point, that perturbing the concentration of Ac∼P may have consequences on growth, remains valid.

**FIG 4:**
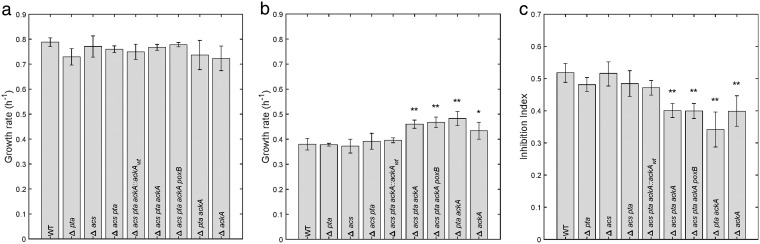
Growth rate of mutant strains in the absence of acetate and in the presence of 128 mM acetate. The growth rate of all mutant strains was measured in a standard shake flask culture as described for Fig. 2 and computed from the data as described in Materials and Methods. We report the means from at least three independent experiments. The error bars represent twice the standard errors of the means. The growth rates without and with acetate are shown in panels a and b, respectively, and the inhibition index is in panel c. The asterisks (* and **) in panels b and c indicate growth rates and inhibition indices for mutant strains that are significantly different from the growth rate and inhibition index of the wild-type strain, for significance thresholds of 0.06 and 0.01, respectively (see Materials and Methods).

The above-described considerations motivate the main question of the present work: do the perturbations of acetate metabolism, due to the inflow of excess acetate from the medium, play a role in growth inhibition by acetate, either by perturbing the distribution of fluxes in central carbon metabolism or by affecting the regulatory role of Ac∼P? In order to answer this question, we constructed mutants that cut different parts of the acetate utilization and synthesis pathways and modify the concentration of Ac∼P. All these mutants are derived from the wild-type strain, BW25113, and their genotypes are listed in Table 1. We measured the growth rate of all strains under our reference conditions (growth on minimal medium with glucose at pH 7.4). As can be seen in Fig. 4a, none of the mutants significantly reduces the growth rate on glucose, in agreement with previous observations for the BW25113 strain under comparable growth conditions (35).

**TABLE 1:** Strains used to dissect different mechanisms of growth inhibition by acetate^*a*^

Strain	Origin
BW25113	Keio collection
BW25113 Δ*ackA*	This study
BW25113 Δ*pta*	This study
BW25113 Δ*pta ackA*	This study
BW25113 Δ*acs*	This study
BW25113 Δ*acs pta*	This study
BW25113 Δ*acs pta ackA*	This study
BW25113 Δ*acs pta ackA poxB*	This study
BW25113 Δ*acs pta ackA*::*ackA_wt_*	This study
BW25113 Δ*actP*	Keio collection
BW25113 Δ*satP*	Keio collection

a All strains were derived from the wild-type strain of the Keio collection, BW25113 (39). This strain is called the wild type. All deletions developed for this study were constructed without leaving antibiotic resistance cassettes on the chromosome. Notice that Δ*pta ackA* is an abbreviation for the Δ*pta* Δ*ackA* double mutant (a similar notation is used for the other strains). The Δ*acs pta ackA*::*ackA_wt_* strain, restoring the original *ackA* gene, was used as a control.

### Δ*ackA* mutant partly relieves growth inhibition by acetate.

We next measured the effect of acetate on the growth rate of the mutant strains by experiments analogous to the one shown in Fig. 2. The results are shown in Fig. 4b. The wild-type and all mutant strains are strongly inhibited by the addition of 128 mM acetate to the growth medium. Even though all strains are strongly affected, we observe two distinct groups: strains that carry the *ackA* deletion grow faster than strains that carry a wild-type copy of the gene. The inhibition indices of Δ*ackA* strains are around 0.4, compared to the values of around 0.5 for all other strains (Fig. 4c). The observed differences in growth rates and inhibition indices, between the wild-type strain on the one hand and the *ackA* mutants on the other, are statistically significant (see Materials and Methods).

To preclude the possibility of unmapped, secondary mutations being responsible for the differential effect in Δ*ackA* strains, we constructed a revertant of the phenotype of the *ackA* deletion. In the Δ*acs pta ackA*::*ackA_wt_* strain, the wild-type allele of *ackA* was restored. The slight growth advantage of the Δ*ackA* strains in acetate, reflected by the smaller inhibition indices (0.4 versus 0.5), disappears in the complemented strain. The inhibition index increases to about 0.47 and reaches the same level as that for the equivalent Δ*acs pta* strain (Fig. 4c). We conclude that even though the construction of the triple mutant has involved many growth and selection steps, the observed phenotypes of the mutants are not due to secondary mutations elsewhere on the chromosome.

The differential effect of the *ackA* deletion is not caused by a decreased flux of acetate into central carbon metabolism (and a corresponding decrease in ATP consumption), since the *pta* deletion, which interrupts the same pathway (Fig. 1), does not reduce growth inhibition by acetate. However, the *ackA* deletion is expected to modify the intracellular concentration of Ac∼P. During growth on glucose without excess acetate, Ac∼P is produced by Pta from acetyl-CoA (23, 36), but in the presence of high concentrations of acetate in the medium, we expect Ac∼P to be mostly generated by phosphorylation of acetate. The latter reaction no longer takes place in a Δ*ackA* strain, so the concentration of Ac∼P will be lower than that in a strain with a functional AckA. In the extreme, deletion of both *ackA* and *pta*, like the Δ*pta ackA*, Δ*acs pta ackA*, and Δ*acs pta ackA poxB* strains shown in Fig. 4, eliminating all reactions that can produce Ac∼P, results in a strain that is completely devoid of Ac∼P (36).

Thus, the 20% decrease of the inhibition index in the Δ*ackA* strains can be explained by (partial) compensation for the increase in Ac∼P expected in the presence of excess acetate in the medium. The growth-inhibitory effect of an increase of the concentration of Ac∼P is consistent with the observation that deleting one of the known deacetylases in E. coli, CobB, reduces the growth rate of E. coli on acetate by almost 2-fold (37). Moreover, in the presence of 160 mM acetate, wild-type cells that are in a growth-arrested state (because of a lack of a nitrogen source in the medium) have a much higher degree of lysine acetylation than the Δ*ackA* derivative (28), suggesting an increased Ac∼P concentration.

So far, we have focused on the reactions producing and consuming acetate inside the cell, ignoring questions about acetate uptake. This is motivated by the fact that, as mentioned in the Introduction, the protonated form of acetate acid, HAc, can freely diffuse into the cell. Nevertheless, two specific transporters of acetate, SatP and ActP, have been identified previously (38). We therefore wanted to ascertain that active acetate uptake or secretion is not necessary for the growth-inhibitory effect of acetate in the medium. Using *satP* and *actP* mutants of the wild-type strain (39), we tested under the same conditions as those described above the effect of adding acetate on the growth rate of the mutant strains compared to that of the wild-type strain. We found no significant difference in the growth inhibition index between the wild type and the mutants (Fig. S4), showing that under our conditions active acetate uptake is not required for growth inhibition.

### Growth inhibition by acetate does not involve reorganization of fluxes in central carbon metabolism.

As explained above, the results reported in Fig. 4 do not support the hypothesis that the influx of excess acetate into central carbon metabolism plays a significant role in growth inhibition. While no such influx can occur in the Δ*acs pta* mutant, in which both the Acs and the Pta-AckA pathways have been eliminated, the growth rate in the presence of acetate and the inhibition index are the same as those for the wild-type strain. In order to further characterize central carbon metabolism on a coarse-grained level (40), we measured the extracellular concentration of glucose and the major fermentation products known to accumulate during aerobic growth of E. coli on glucose in the wild-type strain, in the Δ*acs pta* double mutant, and in the Δ*acs pta ackA* triple mutant. In particular, we quantified acetate, ethanol, formate, lactate, and pyruvate during growth experiments, as described for Fig. 2 (35, 41–43).

**FIG 5:**
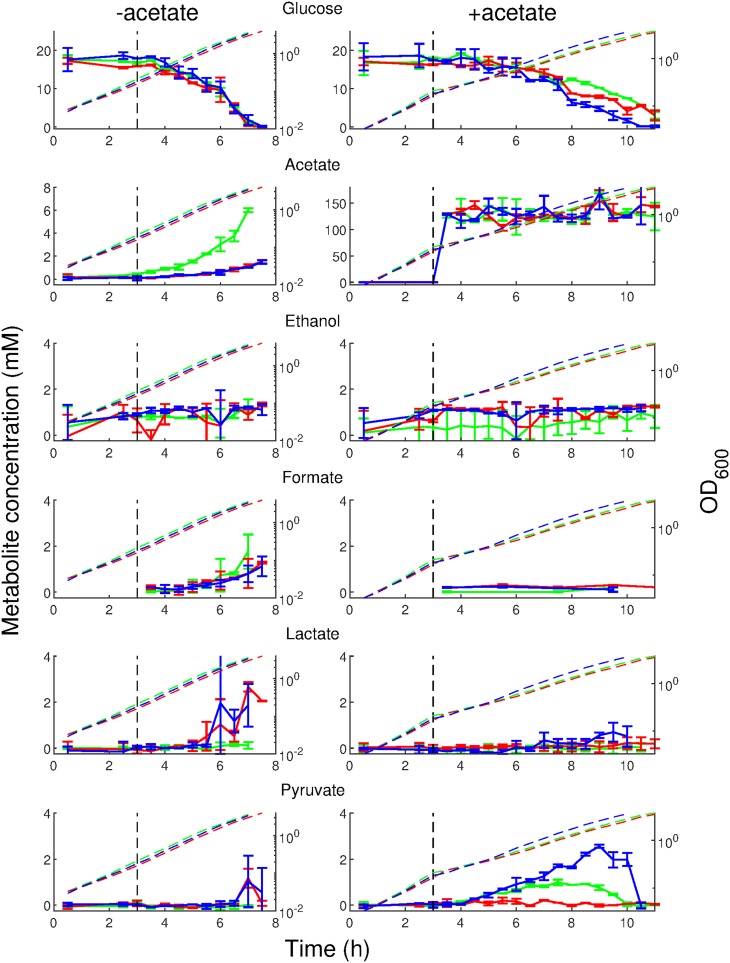
Measurement of metabolites taken up and excreted by E. coli. Bacteria were grown in a shake flask, as described for Fig. 2, and samples were removed at regular time intervals and analyzed for the different metabolites (see Materials and Methods). The left column shows cultures grown on glucose alone. Acetate (128 mM) was added to the cultures in the right column after 3 h of growth (indicated by the vertical dashed line). The metabolite measured is indicated on top of each row. The measurements were carried out in quadruplicate in the wild-type strain (green), the Δ*acs pta* mutant (red), and the Δ*acs pta ackA* mutant (blue). The error bars indicate twice the standard errors of the means. For reference, OD_600_ curves are shown as dashed lines. Note that the ordinate scale is smaller for the metabolites in the bottom four rows, and the ordinate scale of acetate measurements with added acetate is much larger than the others.

The results shown in Fig. 5 (left column) confirm the expected overflow metabolism during growth of the wild-type strain on glucose, namely, the secretion of acetate (31). This overflow metabolism is almost completely abolished in the mutants, confirming that the major pathway of acetate production is interrupted. In order to dissipate the extra reducing equivalents, other metabolites, in particular lactate and pyruvate, are secreted during growth of the mutants on glucose. When 128 mM acetate is added to the growth medium (Fig. 5, right column), the growth rate slows and the glucose uptake rate diminishes accordingly. Because of the lower growth rate, there is no longer any detectable overflow metabolism, except for a small amount of pyruvate in the wild type and triple mutant. Notice that changes in acetate concentration are undetectable, since there is a large excess of acetate in the growth medium under this condition. Diminished pyruvate excretion in the Δ*acs pta* mutant may be an unknown regulatory effect mediated by Ac∼P and affected by the additional deletion of *ackA* in the triple mutant. The slight relief of growth inhibition by acetate in the triple mutant is consistent with the somewhat higher glucose uptake rate in this strain (Fig. 5, right column, blue curve).

Despite some small differences between strains, the data shown in Fig. 5 reveal that the pattern of uptake and secretion of carbon compounds by the cell is not significantly perturbed by the addition of large concentrations of acetate to the growth medium. A similar observation can be made for the changes in extracellular pH. None of the three strains shows a noticeable difference in extracellular pH at a given OD in strains growing in medium with and without added acetate (Fig. S5). Moreover, the extracellular pH curves are quite similar for the wild-type and mutant strains. Using the uptake and secretion patterns as proxies for the functioning of central metabolic pathways, this suggests that addition of acetate indeed does not entail a profound reorganization of metabolism, apart from a global rescaling of fluxes due to the reduced growth rate.

In order to further probe this conclusion, we used a genome-scale reconstruction of E. coli metabolism (29). From the data shown in Fig. 5 we computed the glucose uptake rate and the secretion rates of acetate, ethanol, formate, lactate, and pyruvate, which were integrated into the model to constrain the exchange fluxes. Moreover, the biomass reaction in the model was set equal to the measured growth rate. We also formulated a limited number of additional uptake and reversibility constraints that are directly motivated by the composition of the growth medium and the utilization of glucose as the sole carbon source (see Materials and Methods and File S1).

**FIG 6:**
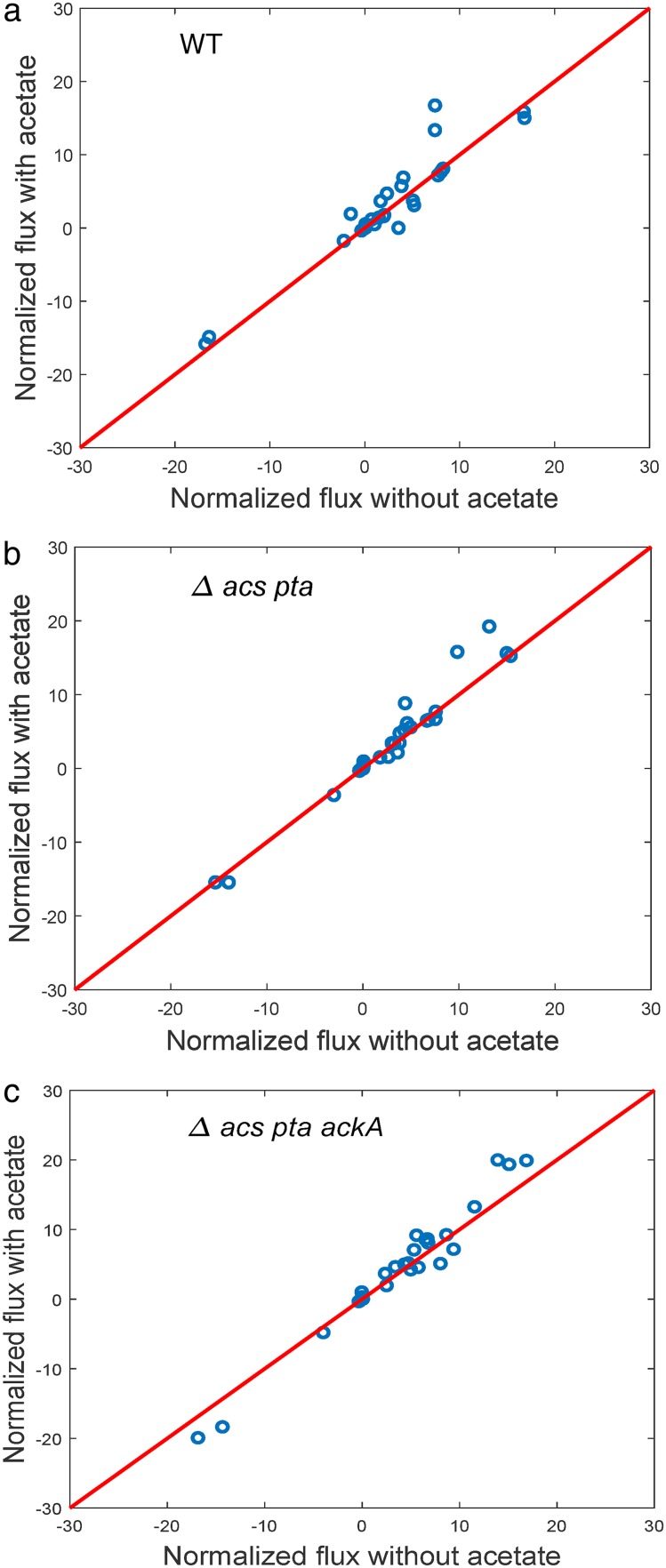
Changes in metabolic fluxes in central carbon metabolism predicted by a genome-scale model of E. coli. The measurements of extracellular metabolites shown in Fig. 5 were used to compute uptake and secretion rates under each of the conditions, as outlined in Materials and Methods. These rates were used to constrain the exchange fluxes of a genome-scale model of E. coli metabolism (29), while the rate of the biomass reaction was set to the experimentally determined growth rate. We used metabolic flux analysis to define a space of solutions consistent with the measured fluxes and the stoichiometry structure of the metabolic network. This solution space was sampled in a random and unbiased manner using a Monte Carlo approach (see Materials and Methods). (a) Scatter plot of the predicted fluxes for 64 reactions in central carbon metabolism of the wild-type E. coli strain growing on glucose, in the absence and presence of acetate. Under each condition, the fluxes have been normalized by the growth rate. The scatter plot shows a very strong correlation between the predicted flux distributions in the absence and presence of acetate, with all reactions clustered around the diagonal (*R*^2^ = 0.89). (b) Same as panel a, except the Δ*acs pta* strain (*R*^2^ = 0.94) was examined. (c) Same as panel a, except the Δ*acs pta ackA* strain (*R*^2^ = 0.94) was examined.

Using this model, we performed a metabolic flux analysis (44, 45) to obtain the flux distribution minimizing the difference between the predicted and measured fluxes, including the biomass production rate (46, 47). We used a Monte Carlo sampling approach to characterize the space of possible solutions, focusing on the reactions in central carbon metabolism (see Materials and Methods). As shown in Fig. 6, when scaling the fluxes under each condition by the growth rate, the distribution of internal metabolic fluxes is the same in the absence or presence of acetate. The results of this computational analysis are in agreement with the conclusions drawn from inspection of Fig. 5 that the inhibitory effect of acetate does not profoundly change the global functioning of metabolism.

As explained above, when 128 mM acetate is added to the medium, quantification of the acetate uptake or secretion rates is unreliable. Therefore, in this case, we did not constrain the corresponding reaction in the metabolic flux analysis. Interestingly, the exchange fluxes predicted by the approach correspond well with the ^13^C flux measurements carried out by Enjalbert et al. (22). In the case of the wild-type strain, the direction of the acetate exchange flux inverts upon the supply of acetate (secretion of 3.5 mmol gDW^−1^ h^−1^ for 0 mM acetate and uptake of 2.7 mmol gDW^−1^ h^−1^ for 128 mM acetate). The deletion of the Pta-AckA pathway, however, prevents this inversion (secretion of 0.7 mmol gDW^−1^ h^−1^ for 0 mM acetate and secretion of 3.8 mmol gDW^−1^ h^−1^ for 128 mM acetate).

## DISCUSSION

The question of which molecular mechanisms underlie growth inhibition of Escherichia coli cultures by excess acetate in the growth medium is of fundamental interest for understanding the physiology of this bacterium but may also have important implications for applications in food preservation and biotechnology. The potential mechanisms explaining the observed growth inhibition have been debated for many decades (48). The classical hypotheses put forward to explaining growth inhibition by acetate are the uncoupling effect of weak acids and the perturbation of the anion concentration caused by the accumulation of acetate anions in the cell (15, 17, 18, 20, 30, 49). Recent work has shown that acetate, when present at high concentrations in the medium, can be assimilated by E. coli even when growing on glucose (22). This suggests that a net uptake of acetate through the AckA-Pta pathway could perturb the fluxes in central metabolism necessary for sustaining maximal growth. Moreover, it could affect the concentration of Ac∼P, an intermediate of the AckA-Pta pathway known to assume a wide range of regulatory functions in the cell (23, 24).

In this work, we have focused on the latter hypothesis. In order to investigate the role of acetate metabolism in growth inhibition by acetate, we have developed a series of E. coli mutant strains that probe relevant parts of the metabolic pathways of acetate synthesis and consumption. In particular, we constructed mutant strains that prevent external acetate from being metabolized by the cell by deleting both the Acs and the Pta-AckA pathways. Within the Pta-AckA pathway, we can allow the production of Ac∼P by deleting just one of the genes or prevent all synthesis of Ac∼P by deleting both genes, thereby probing potential regulatory roles of this metabolite. For several of the mutants we have measured growth rates and extracellular concentrations of a number of by-products of central carbon metabolism, known to accumulate in the growth medium in wild-type E. coli strains and strains with deletion of the *pta*, *ackA*, or *acs* gene. All measurements have been carried out under carefully controlled reference conditions, which allow crossing and comparing the results obtained for the different E. coli strains. However, some care should be exercised in generalizing the results to other organisms and conditions.

By means of these strains, we tested the hypothesis that the influx of excess acetate in the medium overloads the metabolic pathways of acetate utilization and thereby perturbs central carbon metabolism. This hypothesis is clearly not supported by our data: (i) the deletion of both *pta* and *acs* prevents acetate utilization by the bacteria but in no way relieves growth inhibition (Fig. 4), and (ii) the functioning of central carbon metabolism in the wild type and Δ*acs pta* mutant are not greatly perturbed by the addition of acetate to the growth medium (Fig. 5). These conclusions are corroborated by integrating the data with a genome-scale model of E. coli metabolism to predict the possible intracellular flux distributions consistent with the measured uptake and secretion rates and growth rate. The analysis indicates that when a scaling factor due to the growth rate difference is accounted for, the predicted distributions of internal fluxes before and after acetate addition are essentially the same (Fig. 6). This suggests that the net influx of excess acetate into central carbon metabolism does not produce a suboptimal flux distribution responsible for the reduced growth rate.

As explained above, the utilization of acetate may also perturb metabolic functioning in a different way, by the phosphorylation of acetate to Ac∼P by AckA and the Ac∼P-mediated modification of enzyme activity. Ac∼P contains a high-energy bond between phosphate and the acetyl moiety and can therefore transfer either the phosphate group to an appropriate acceptor, in this case two-component response regulators, or the acetyl group to lysine of target proteins. Since the numbers of targets of both regulatory mechanisms are in the hundreds or thousands (23, 24, 26, 27), we cannot individually assess all these interactions. However, we can measure the global effect on growth rate by preventing the production of Ac∼P. Our results show that in the Δ*acs pta ackA* triple mutant, a strain devoid of Ac∼P, the addition of acetate has a slightly weaker effect on growth. We therefore conclude that part of the growth-inhibitory effect of acetate seems to involve the perturbation of Ac∼P levels in the cell, thereby interfering with the regulatory role of this signaling metabolite. From our data, we estimate that this accounts for about 20% of the observed reduction in growth rate.

The question that immediately comes up is what accounts for the remaining 80% of the reduction in growth rate. We mentioned two commonly advanced hypotheses in the Introduction. First, the classical explanation of growth inhibition by acetate and other weak acids is uncoupling (15–17). Acetic acid, HAc, diffuses into the cell, where it dissociates into acetate, Ac^−^, and a proton, H^+^. In order to maintain the membrane potential, the protons need to be pumped out of the cells, which costs ATP and thus draws away energy from growth. Another explanatory hypothesis involves the perturbation of the anion composition of the cell, leading to the inhibition of enzyme activity by the accumulating Ac^−^ anions themselves or by the replacement of pools of other anions regulating enzyme activity. It has been shown previously that acetate inhibits methionine biosynthesis and, more particularly, the activity of the MetE enzyme (20, 33). While there is no evidence that acetate acts directly on the enzyme, it is very possible that enzyme inhibition is mediated by a change in concentration of another anion following acetate addition to the medium. Moreover, acetate may act on the transcription of enzymatic genes, as found in a recent study (50).

**FIG 7:**
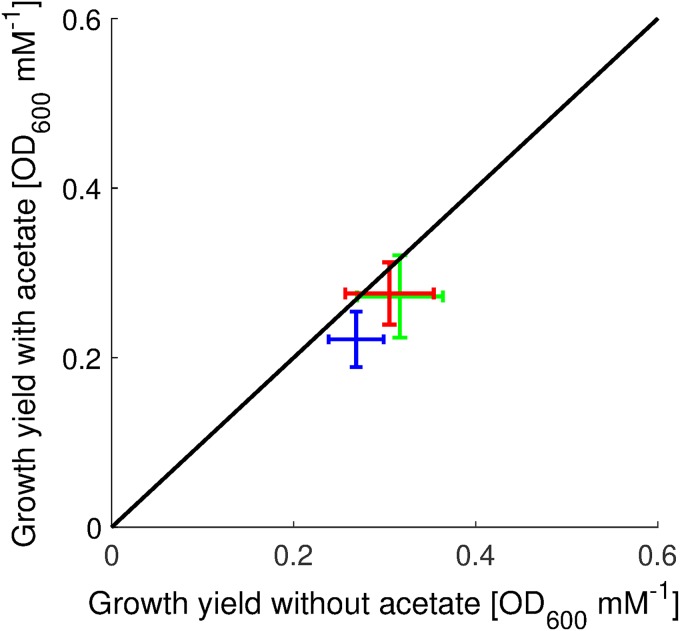
Biomass yield of wild-type mutant strains in the absence of acetate and in the presence of 128 mM acetate. The biomass yields of the wild-type strain (green), the Δ*acs pta* mutant (red), and the Δ*acs pta ackA* mutant (blue), in the absence of acetate and in the presence of 128 mM acetate, were measured as described in Materials and Methods. We report the means from four independent experiments. The error bars represent twice the standard errors of the means. The diagonal corresponds to identical biomass yields in the presence and absence of acetate. Although the measurement means are located below the diagonal, suggesting a lower biomass yield in the presence of acetate, the pairwise differences in yield with and without acetate are not statistically significant at confidence levels of 0.01 and 0.06 (see Materials and Methods).

Our study does not provide a definite answer to the question of which of the two effects identified above is (mainly) responsible for growth inhibition by acetate. However, some of our observations argue against the uncoupling hypothesis. First, if uncoupling played an important role, one would expect the biomass yield to be significantly lower in cultures growing in the presence of high concentrations of acetate in the medium, reflecting the energy-spilling activity of the proton pumps (19). Estimating the yields from the data shown in Fig. 5 gives a somewhat different result. While biomass yields in the presence of acetate are slightly higher than those in the absence of acetate, in the sense that the measurements are located below the diagonal of the scatterplot in Fig. 7, the differences are too small (<15%) to be statistically significant for the given measurement uncertainties. Moreover, their effect seems too weak to account for the strong reduction in growth rate observed under our conditions. These observations are consistent with previous reports (19, 22).

Second, it should be emphasized that the uncoupling hypothesis posits a futile cycle in which not only H^+^ but also Ac^−^ molecules are pumped out of the cell following the diffusion of HAc into the cell (15). This necessarily involves active transport of acetate. Two acetate transporters have been reported in the literature, SatP and ActP, and the deletion of either of these was shown to halve the acetate uptake rate (38). Given the high rate of acetate secretion necessary for obtaining a significant reduction in growth rate, one would expect that deleting either SatP or ActP would reduce the flux through the futile cycle and, thus, energy spilling and growth inhibition. The results reported in the section “*ΔackA* mutant partly relieves growth inhibition by acetate,” above, and in Fig. S4 in the supplemental material do not confirm this. Growth inhibition is as strong in the mutants as in the wild type.

The above-described arguments are suggestive but need to be supported by a quantitative characterization of the membrane potential and other energetic variables as well as a precise carbon balance in order to unambiguously rule out an important role for uncoupling. Previous studies have shown the occurrence of an anion imbalance in the presence of high concentrations of Ac^−^ in the cytoplasm, for example, a 6-fold decrease in glutamate concentration (18). We found that under our conditions, (partial) relief of growth inhibition by methionine is small (Fig. S2), contrary to previous studies (18), although this does not exclude that the perturbed anion balance affects other enzymes, as discussed above. While the cumulative effect of a modified anion distribution on specific reactions may well constitute a major factor of growth inhibition by acetate, only global metabolic studies correlating flux distributions with anion concentrations will be capable of identifying all reactions that are sensitive to specific anions. Although our study does not provide a definite answer to the question of what causes growth inhibition by acetate and other weak acids in bacteria, it does uncover a new regulatory effect by Ac∼P and identifies promising directions in which to further investigate other possible explanations.

## MATERIALS AND METHODS

### Bacterial strains and growth media.

The bacteria used in this study were E. coli K-12, strain BW25113 (39), that we designated the wild type (*rrnB_T14_* Δ*lacZ_WJ16_ hsdR514* Δ*araBAD_AH33_* Δ*rhaBAD_LD78_*). The following deletion mutants were constructed by removing the entire open reading frames of the corresponding genes: the Δ*acs*, Δ*ackA*, Δ*pta*, Δ*pta ackA*, Δ*acs pta*, Δ*acs pta ackA*, and Δ*acs pta ackA poxB* strains. Δ*pta ackA* is the shorthand notation for the Δ*pta* Δ*ackA* double mutant (an analogous abbreviation is used for the other strains). We also constructed a Δ*acs pta ackA*::*ackA_wt_* reversion mutant. In this mutant, a wild-type copy of the *ackA* gene was reintroduced into the mutant strain in order to verify the strain constructions. The phenotype of the resulting complemented strain should be identical to that of the Δ*acs pta* strain.

The standard minimal medium contained 11.1 mg/liter CaCl_2_, 240.73 mg/liter MgSO_4_, 5 mg/liter thiamine, 1 g/liter NH_4_Cl, 0.5 g/liter NaCl, 3 g/liter KH_2_PO_4_, 8.5 g/liter Na_2_HPO_4_·2H_2_O, 3 mg/liter FeSO_4_·7H_2_O, 15 mg/liter Na_2_EDTA·2H_2_O, 4.5 mg/liter ZnSO_4_·7H_2_O, 0.3 mg/liter CoCl_2_·6 H_2_O, 1 mg/liter MnCl_2_·4H_2_O, 1 mg/liter H_3_BO_3_, 0.4 mg/liter Na_2_MoO_4_·2H_2_O, and 0.3 mg/liter CuSO_4_·5H_2_O. In order to obtain a growth medium at pH 7.4 or 6.4, the relative concentrations of KH_2_PO_4_ and Na_2_HPO_4_·2H_2_O were adjusted appropriately without changing the total (molar) phosphate concentration. As a carbon source, 3 g/liter glucose was used. The growth medium was supplemented with methionine to a final concentration of 3.3 mM when appropriate. Acetate was added to the growth medium as a concentrated solution of sodium acetate equilibrated to pH 7.4 or pH 6.4 in order to obtain the desired final concentration of acetate (128 mM in most experiments).

### Construction of E. coli mutants.

All of our mutants were derived from strains in the Keio collection (39). The kanamycin resistance cassette replacing the coding sequence of the genes was removed such that none of our mutants carries an antibiotic resistance cassette. The kanamycin resistance cassette is flanked by recognition sites of the Flp recombinase, and the cassette can therefore be excised using a plasmid expressing the Flp recombinase (plasmid 705-FLP) (51). This excision creates an in-frame scar sequence (102 bp), reducing polar effects on downstream gene expression. The first mutants to be constructed, by simply removing the kanamycin resistance cassette from the corresponding Keio clone, were the Δ*ackA* and Δ*pta* mutants.

We constructed the Δ*acs* and the Δ*pta ackA* mutants by replacing the gene *acs* and the operon *pta-ackA* with an FLP recombination target-flanked kanamycin resistance gene generated by PCR (52). Primers have 20-nucleotide (nt) 3′ ends homologous to the kanamycin resistance cassette used in the Keio collection and 50-nt 5′ ends of homology targeting the chromosomal region of interest. PCR products were transformed into a BW25113 strain expressing the λ Red recombinase (plasmid pSIM5) (53). Antibiotic-resistant recombinants were then selected and the kanamycin resistance cassette removed.

We constructed the Δ*acs pta*, Δ*acs pta ackA*, and Δ*acs pta ackA poxB* mutants by P1 transduction (54). The P1 lysate was grown on our Δ*pta ackA*::*kan* mutant and the Δ*pta*::*kan* mutant from the Keio collection. These lysates were then used to infect the Δ*acs* strain in order to obtain Δ*acs pta ackA*::*kan* and Δ*acs pta*::*kan* transductants. The kanamycin resistance cassette was removed as described above. The same procedure was used for moving the Δ*poxB*::*kan* mutation from the Δ*poxB* Keio mutant to our Δ*acs pta ackA* strain.

We reintroduced the gene *ackA* into the Δ*acs pta ackA* mutant precisely into the original locus by following a previously described approach (55), thereby effectively restoring a Δ*acs pta* mutant. Primers pta-CCDB1 and ackA-KN1 were used to amplify a *kan*:p_BAD_:*ccdB* cassette. The PCR product was transformed into a Δ*acs pta ackA* mutant expressing the λ Red recombinase (plasmid pSIM5). Antibiotic-resistant recombinants were selected. Primers Y2-ACKA and ackA_pta_left_PCR_verif were used to amplify the sequence between the initiation codon of *ackA* and the initiation codon of *pta* of the Δ*acs pta* mutant. The PCR product was recombined into the chromosome in place of the cassette. Recombinants were selected on medium containing arabinose for activation of the suicide gene *ccdB*, which kills cells that have not recombined the *ackA* gene.

All mutants were verified by PCR and DNA sequencing. The list of primers used in this study can be found in Table 2.

**TABLE 2:** Oligonucleotides used in this study

Primer name^*a*^	Sequence (5′ to 3′)^*b*^	Purpose
acs1P1	GTTACCGACT CGCATCGGGC AATTGTGGGT TACGATGGCA TCGCGATAGC ATTCCGGGGA TCCGTCGACC	Construction
acs2P2	AACGCTTATG CCACATATTA TTAACATCCT ACAAGGAGAA CAAAAGCATG TGTAGGCTGG AGCTGCTTCG	Construction
acs-Right-PCR-verif	AAAACTGCCA ATACCCCT	Verification
acs-Left-PCR-verif	TTTTAATTCC CGCTCCCT	Verification
ackA-pta-Left-Primer	TGGCTCCCTG ACGTTTTTTT AGCCACGTAT CAATTATAGG TACTTCCATG ATTCCGGGGA TCCGTCGACC	Construction
ackA-pta-Right-Primer	GCAGCGCAAA GCTGCGGATG ATGACGAGAT TACTGCTGCT GTGCAGACTG TGTAGGCTGG AGCTGCTTCG	Construction
ackA-pta-Left-PCR-verif	CCCTGACGTT TTTTTAGCC	Verification
ackA-pta-Right-PCR-verif	CAGCGCAGTT AAGCAAGA	Verification
ackA-Right-PCR-verif	TATCCTCTTT CGTTACCGCC	Verification
pta-Left-PCR-verif	GGCGGTAACG AAAGAGGA	Verification
poxB-seq-Rev	CTCCTTTCTC TCCCATCCC	Verification
poxB-seq-Fwd	TAAACGTCGT CCCCAACC	Verification
pta-CCDB1	CTTTCTAGAG AATAGGAACT TCGAACTGCA GGTCGACGGA TCCCCGGAAT TTATATTCCC CAGAACATCA GG	Construction
ackA-KN1	TGGCTCCCTG ACGTTTTTTT AGCCACGTAT CAATTATAGG TACTTCCatg ATAGGAACTT CAAGATCC	Construction
Y2-ACKA	CTTTCTAGAG AATAGGAACTT CGAACTGCAG GTCGACGGAT CCCCGGAATc acGGTTTATC CTCTTTCGT	Construction
ackA compl verif	CGCAAAATGG CATAGACTCA A	Verification

a Note that ackA-Right-PCR-verif and ackA-pta-Left-PCR-verif were used to verify the *ackA* deletion, pta-Left-PCR-verif and ackA-pta-Right-PCR-verif were used to verify the *pta* deletion, and ackA-pta-Right-PCR-verif and ackA-compl-verif were used for verifying the *ackA* complementation.

b The lowercase sequences in ackA-KN1 and Y2-ACKA represent the start codons of the genes *ackA* and *pta*, respectively.

### Growth in shake flasks.

For each strain, a seed flask (50-ml capacity), containing 10 ml of filtered minimal medium with glucose, was inoculated from a glycerol stock. The culture in the seed flask was grown overnight at 37°C with orbital agitation of 200 rpm. At the same time, 50 ml of filtered minimal medium with glucose (and methionine when appropriate) was pipetted into different 250-ml flasks (as many as there were experimental conditions and replicates) and stored overnight at 37°C without shaking. The following day, each 250-ml flask was inoculated to an OD_600_ of 0.02 from the seed flask. For each strain, two 250-ml flasks were used: one for the addition of 2 ml of filtered minimal medium with acetate and the other for the addition of 2 ml of filtered minimal medium without any carbon source (control). Cultures were grown at 37°C with orbital shaking at 200 rpm. Growth of the strains was monitored every 30 min by removing a sample of 1 ml. Samples were used to measure the optical density. The remaining volume was centrifuged at 14,000 × *g* for 3 min at 4°C. The supernatant was frozen at −20°C for the quantification of metabolites. Acetate stock solution was prepared in concentrated form such that 2 ml of the stock solution, added to the culture, would give a final concentration of 128 mM acetate. Minimal medium with acetate and minimal medium without any carbon source were stored at 37°C before addition to the growing culture. Acetate was added when the OD_600_ reached about 0.2.

### pO_2_, pH, and OD measurements.

Cell growth was monitored by measuring the OD_600_ with a spectrophotometer (Eppendorf BioPhotometer). Dilutions were done when appropriate in order to stay in the range of linearity of the instrument. The partial oxygen pressure, pO_2_, and the pH were measured with a Clark electrode (LAMBDA fermentor) and a pH (micro)probe (Mettler Toledo or Thermo Scientific Orion), respectively.

### Quantification of metabolite concentrations in the medium.

d-Glucose, acetic acid, formic acid, pyruvic acid, d-lactate, and ethanol were assayed by enzymatic assay kits according to the manufacturer’s recommendations: R-Biophar no. 10 716 251 035 (Boehringer Mannheim), K-ACETRM (Megazyme), K-FORM (Megazyme), K-PYRUV (Megazyme), K-LATE (Megazyme), and K-ETOH (Megazyme), respectively. All of the above-mentioned measurement procedures are based on coupled enzyme assays.

Quantifications were done in 96-well microplates (clear, flat bottomed, plastic). Depending on the metabolite we wanted to quantify, different enzymatic reactions led to the consumption or the production of NADH. The concentration change of NADH was quantified by measuring the difference in absorbance at 340 nm (Δ*A*_metabolite_) with a microplate reader (Perkin Elmer Fusion Alpha). The concentration of the sample, *C*_metabolite_ (diluted in order to remain within the linearity region of the assay) is then calculated as

(2) $$C_{\text{metabolite}}=\frac{\Delta A_{\text{metabolite}}}{\Delta A_{\text{standard}}}\cdot C_{\text{standard}}$$

where Δ*A*_standard_ and *C*_standard_ are the measured absorbance difference and the concentration of the metabolite standard. The metabolite standard solution was provided with each kit. In order to compute Δ*A*_metabolite_ and Δ*A*_standard_, the absorbance before starting the reactions (*A*_1_) and the absorbance at the end of the reactions (*A*_2_) were read ten times at regular time intervals in order to ensure that the reaction had reached equilibrium. In order to compensate for drift in the measurements, we fitted a straight line to the repeated measurements of *A*_1_ and *A*_2_. Using this straight-line extrapolation, the absorbance difference, Δ*A* = *A*_2_ – *A*_1_, was calculated at the time of addition of the last enzyme that starts the reactions. Metabolite concentrations were corrected to take into account the dilution due to the addition of 2 ml of medium with or without acetate. Concentrations are given as the means from at least three independent experiments. Error bars are set equal to twice the standard errors of the means.

### Estimation of growth rates and uptake and secretion rates.

In order to compute growth rates for the different strains, cultured in the presence or absence of acetate, we used the exponential growth model

(3) $$\frac{d}{\mathrm{dt}}B\left(t\right)=\mu \cdot B\left(t\right),B\left(0\right)=B_{0}$$

with *B*(*t*) and *B*_0_ being the time-varying and initial biomass in OD_600_ units, respectively, and *μ* the growth rate (per hour). This model has the explicit solution

(4) $$B\left(t\right)=B_{0}\cdot e^{\mu t}$$

This equation was fitted to each individual time series of optical density measurements. We checked that within the chosen time interval, the underlying assumption of exponential growth at a constant rate is satisfied. The reported growth rate values are the means from at least three independent experiments. Error estimates are reported as twice the standard errors of the means.

In order to compute the glucose uptake rate, we combined the growth model with the glucose consumption model

(5) $$\frac{d}{\mathrm{dt}}G\left(t\right)=-r_{\text{glc}}\cdot B\left(t\right),G\left(0\right)=G_{0}$$

which has the explicit solution

(6) $$G\left(t\right)=G_{0}-\left(B_{0}/Y\right)\cdot \left(e^{\mu t}-1\right)$$

where *G*(*t*) and *G*_0_ are the time-varying and initial glucose concentrations (in millimolars), respectively, and *Y* (OD_600_ per millimolar) is the biomass yield, defined as the ratio of the growth rate and the glucose uptake rate, *r*_glc_ (in millimolars per OD_600_ unit per hour). We simultaneously fitted equations 4 and 6 to each individual time series data set of glucose concentrations and optical densities, obtained in a single growth experiment. For each of the six conditions considered (wild-type, Δ*acs pta*, and Δ*acs pta ackA* strains under the two growth conditions, 0 or 128 mM acetate added to the glucose minimal medium), estimates of *μ* and *Y* were obtained. The glucose uptake rate can be directly obtained from these estimates, bearing in mind that *Y* = *μ*/*r*_glc_. The reported values are the means from four independent replicate experiments. Error estimates are reported as twice the standard errors of the means.

In order to obtain the secretion rate of the fermentation by-products, used in the flux balance model, we again fitted equation 6 to the data but replaced the glucose uptake rate with the appropriate secretion rate and used the values of *μ* and *B*_0_ obtained as described above. All uptake and secretion rates are computed from four independent replicate experiments with error estimates given by twice the standard errors of the means.

### Metabolic flux analysis.

All computational analyses were performed with a slightly modified version of the genome-scale reconstruction iAF1260-flux1 of Escherichia coli metabolism (29), to which we added a reaction accounting for transport of acetate anions by ActP (38). The lower bound of exchange fluxes was set to zero, except for components of the *in silico* growth medium (water, vitamin, salts, traces, and glucose), which were left unconstrained, and for the oxygen uptake rate, which was limited to 20 mmol gDW^−1^ h^−1^. The upper bound of the exchange fluxes was set to zero for secreted products, except for those detected in the external medium in our experiments, namely, acetate, formate, lactate, pyruvate, and ethanol. These fluxes were set to their measured values ± two standard errors of the means, except for acetate when excess acetate is supplied to the medium. A theoretical exchange flux of carbon dioxide was determined based on the carbon mass balance. Eighteen additional reactions were constrained by literature data to allow normal functioning of the glycolysis and the pentose phosphate pathway (File S1). Reactions allowing glycogen consumption and transport of d-glucose through alternative pathways were blocked, as were fluxes through reactions catalyzed by putative sugar phosphatase and aldehyde dehydrogenase. The maintenance fluxes were set to their default values (59.81 mmol gDW^−1^ h^−1^ and 8.39 mmol gDW^−1^ h^−1^ for the growth- and non-growth-associated maintenance fluxes, respectively). We checked that this allows flux balance analysis to reproduce the measured growth rate of the wild-type strain cultured in minimal medium with glucose in the absence of acetate, when the objective function is the maximization of biomass. The biomass function used is Ec_biomass_iAF1260_core_59p81M (29).

In order to test the consistency of the metabolite measurements with the network stoichiometry, we performed a metabolic flux analysis (44), where the objective is to minimize the differences between the measured and predicted exchange fluxes and growth rate. Let *v* denote the vector of fluxes at steady state, *N* the stoichiometry matrix, *v^l^* and *v^u^* the vectors of upper and lower bounds on fluxes, respectively, and v^ the vector of *p* measurements of exchange fluxes. We assume that the first *p* elements of *v* correspond to the measured exchange fluxes. Moreover, we define *u*^+^ and *u*^–^ as nonnegative dummy variables. Following the formulation of reference 46 (see also references 45 and 47), metabolic flux analysis can be formulated as the following linear programming problem:

$$\min\overset{p}{\underset{j=1}{\sum }}\left(u_{j}^{+}+u_{j}^{-}\right)subjectto$$

N·v=0,

$$v^{l}\leq v\leq v^{u},$$

vj−uj++uj−=v^j,forallj=1,…,p,

$$u^{+},u^{-}\geq 0$$

This minimization problem was solved for each of the strains considered, both in the absence and presence of acetate, using the COBRA v3.0 Toolbox (56) with Gurobi 7.5.2 as the linear programming solver (Gurobi Optimization, Inc., Houston, TX).

In order to further characterize the solution space of the above-described metabolic flux analysis problem, we used a Monte Carlo sampling approach to estimate for each reaction in the network a distribution of possible flux values (57). In particular, we performed uniform random sampling by means of the coordinate hit-and-run with rounding (CHRR) algorithm implemented in the COBRA Toolbox (58). We computed the distribution of reaction fluxes consistent with the measured growth rate and exchange fluxes for each condition, focusing on reactions in central carbon metabolism. These reactions were determined by their annotation in the iAF1260-flux1 model: pentose phosphate pathway, anaplerotic reactions, glycolysis gluconeogenesis, pyruvate metabolism, and citric acid cycle. The maximum of the resulting distributions, one for each reaction, was used for comparing reaction fluxes in the presence or absence of acetate in the growth medium. To this end, the reaction fluxes were rescaled by dividing them by the measured growth rate.

### Quantification of Acs expression.

In order to quantify Acs expression, we used a fluorescent reporter gene system based on a transcriptional fusion of the *acs* promoter with a stable green fluorescent protein, GFPmut2, carried on the low-copy-number plasmid pUA66 (59). The wild-type strain was transformed with this plasmid, and experiments were carried out under the reference conditions described above. In addition to being used for measuring the optical density, samples taken were transferred to a 96-well plate to quantify the fluorescence emitted by the cultures in a microplate reader (Tecan Infinite 200 PRO). The excitation wavelength was set to 480 nm, and the emitted fluorescence was measured at 520 nm.

The data were corrected for background fluorescence levels using the wild-type strain as described previously (60). An estimate of the reporter protein concentration, in arbitrary units, was obtained by dividing, at each time point, the background-corrected fluorescence level by the optical density. Assuming that Acs is a stable protein, like the GFP variant used in this study, the reporter concentration can be assumed to be proportional to the Acs concentration (61).

### Statistical tests.

In order to test the hypothesis that the growth rates or biomass yields of two strains in a given condition are equal, we used Welch’s *t* test, a *t* test for normally distributed variables with possibly unequal variances and an unequal number of independent samples (experiments) (62). A pair of strains failing the test, for significance thresholds of 0.06 or 0.01, are concluded to have significantly different growth rates.

No off-the-shelf statistical method is available for testing the hypothesis that two inhibition indices are equal, as inhibition indices, which quantify the effect of acetate on the growth rate, are defined as ratios of normally distributed variables with nonzero means (equation 1). We therefore followed a bootstrap procedure by randomly resampling with replacement the experimentally determined distributions of the growth rates and computing the corresponding inhibition indices, thereby obtaining estimates of the mean and confidence interval of the inhibition indices (63). These bootstrap distributions were used to test the null hypothesis that the inhibition indices of two strains under a given condition are equal by computing the *P* value corresponding to the probability that the difference between the inhibition indices estimated from the data would occur if the two indices were equal. The inhibition indices were concluded to be different for *P* values below 0.03 or 0.005, corresponding to significance thresholds of 0.06 or 0.01, respectively, because of the two-sidedness of the distribution.

## Supplementary Material

Supplemental file 1

Supplemental file 2
