# Supplementary material for: Acetate Metabolism and the Inhibition of Bacterial Growth by Acetate
Source: J Bacteriol. 2019 Jun 10;201(13):e00147-19. doi: 10.1128/JB.00147-19 (PMC6560135; doi:10.1128/JB.00147-19)
Supplement: Supplemental file 2 [file JB.00147-19-s0002.pdf]

Supplementary information to 'Acetate metabolism and the inhibition of bacterial growth by acetate'

Stéphane Pinhal, Delphine Ropers,
Johannes Geiselmann, and Hidde de Jong

Supplementary Figure 1: Growth characteristics of strain BW25113 grown in minimal glucose medium

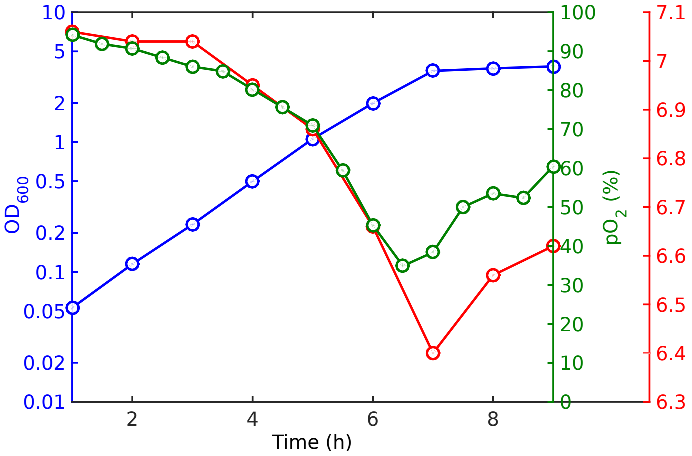

The curves show the main parameters characterizing the growth of this *E. coli* strain: optical density in blue, oxygen pressure in green, and pH of the growth medium in red. The cells grow exponentially for about 7 hours after inoculation. The growth characteristics are identical to profiles measured for similar strains of *E. coli* [1].

Supplementary Figure 2: Effect of methionine on growth inhibition by acetate

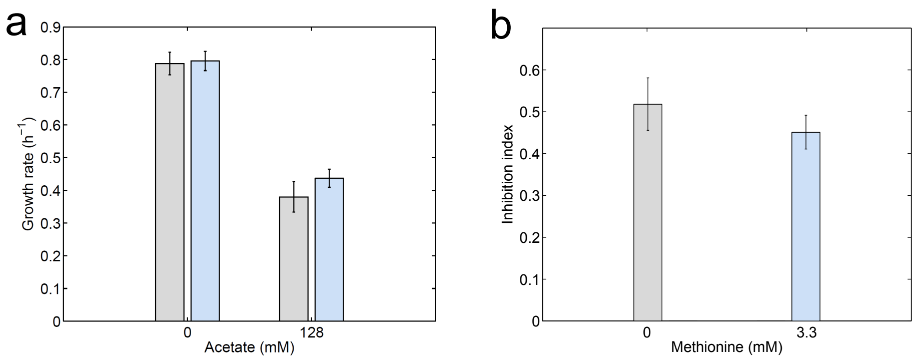

(a) The growth rate of the wild-type strain in the absence (gray) or presence (blue) of 3.3 mM methionine was measured as described in the *Materials and methods*. The addition of acetate is indicated below the bar graph. The error bars represent twice the standard error of the mean of at least three independent experiments. (b) Bar graph representing the same data in terms of the inhibition index. For the given measurement uncertainties, neither the growth rates nor the inhibition index are significantly different in the presence as compared to the absence of methionine (*Materials and methods*).

Supplementary Figure 3: Expression of Acs during growth inhibition by acetate

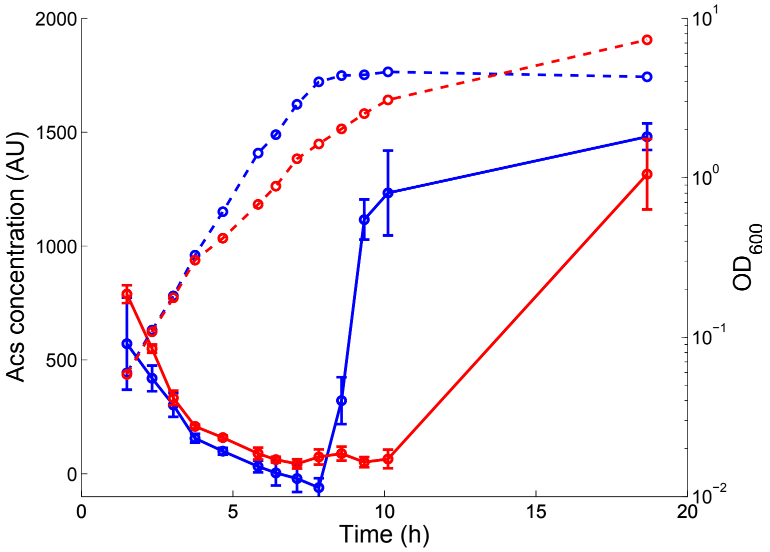

Expression of the gene *acs* was monitored during a flask experiment in the reference conditions, minimal medium with glucose at pH 7.4, adding a solution without acetate (blue curves) or with 128 mM acetate (red curves) after 4 h of growth (*Materials and methods*). The (solid) curves show estimates of the Acs concentration, in arbitrary units, obtained by dividing at each time-point the fluorescence intensity by the optical density (*Materials and methods*). For reference, the optical density is shown as well (dashed curves). We report the mean of at least three indepedent experiments. The error bars represent twice the standard error of the mean. Acs present in the cells from the preculture is diluted out to a low concentration after inoculation, whether growth inhibition due to acetate addition occurs or not. Acs starts to be synthesized significantly only after glucose exhaustion.

Supplementary Figure 4: Growth rate of acetate transporter mutant strains in the absence of acetate and in the presence of 128 mM acetate

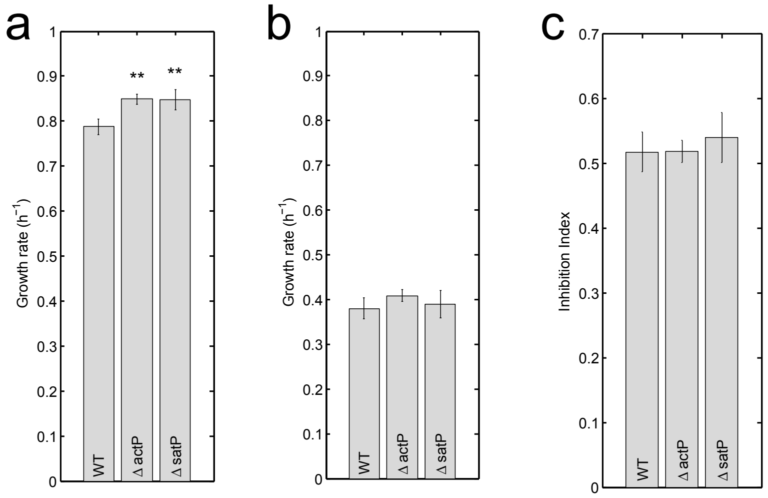

The growth rate of the *satP* and *actP* deletion mutant strains was measured in a standard shake flask culture as in Figure 2 and computed from the data as described in the *Materials and Methods*. We report the mean of four independent experiments. The error bars represent twice the standard error of the mean. The growth rate without and with acetate are shown in (a) and (b), respectively, and the inhibition index in (c). The growth rate of the mutant strains in the absence of acetate is slightly but significantly higher (for a significance threshold of 0.01, see *Materials and methods*), as indicated by the asterisks **. None of the differences in the inhibition indices are statistically significant, however, due to the fact that the growth rates of the mutants are also marginally higher in the presence of acetate. This shows that the magnitude of the growth-inhibitory effect is the same in the wild-type and in the acetate transporter mutant strains.

Supplementary Figure 5: Quantification of pH in wild-type and mutant strains in the absence of acetate and in the presence of 128 mM acetate

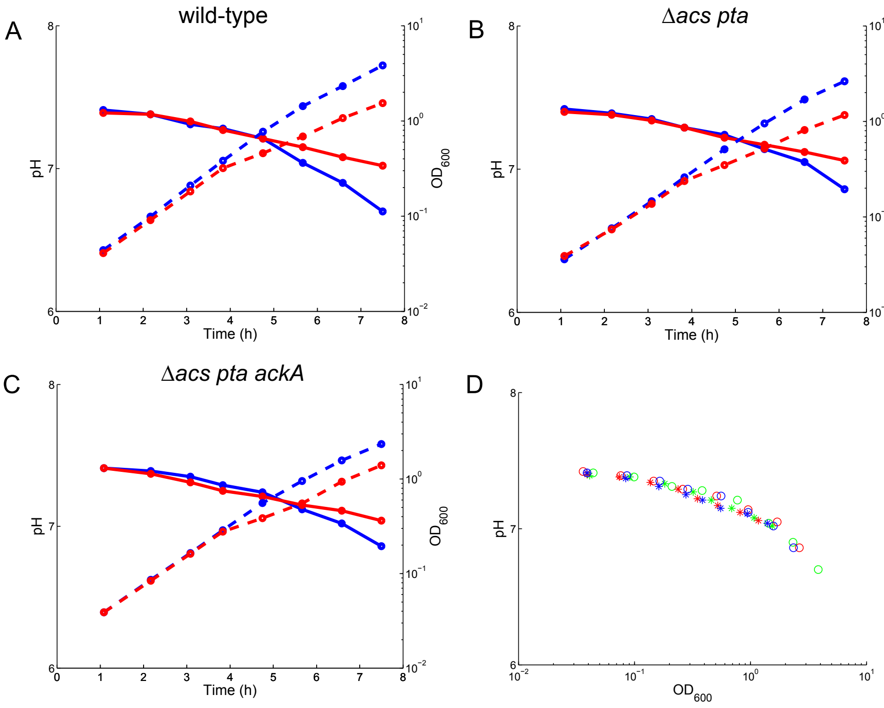

Bacteria were grown in a shake flask, as described in Figure 2, and samples were removed at regular time intervals to measure OD_{600} and pH levels (*Materials and methods*). (a) Measurements of pH (solid curve) and OD_{600} (dashed curve) for the wild-type strain in absence (blue) and presence (red) of 128 mM acetate, added after approximately 4 h of growth. (b) Idem for the Δ*acs pta* strain. (c) Idem for the Δ*acs pta ackA* strain. (d) Plot of the relation between pH and OD_{600}, combining the data from panels (a)-(c). The measurements for the wild-type strain are shown in green, for the Δ*acs pta* strain in red, and for the Δ*acs pta ackA* strain in blue. The measurements without addition of acetate are marked by 'o' and those with acetate by '*'. In all strains, the pH decreases with increasing OD_{600}. This relation is very much the same for strains grown in the absence and presence of acetate.

Supplementary File 1: Additional uptake and reversibility constraints for metabolic flux analysis

The file contains additional uptake and reversibility constraints used in the metabolic flux analysis. The constraints are directly motivated by the composition of the growth medium, the utilization of glucose as the sole carbon source, and the deletions in the strains. The tabs in the file list the constraints for the three strains (wild-type, Δ*acs pta* mutant, and Δ*acs pta ackA* mutant) and the two conditions (0 and 128 mM acetate) considered.

References

[1] Brice Enjalbert, Fabien Letisse, and Jean-Charles Portais. Physiological and molecular timing of the glucose to acetate transition in *Escherichia coli*. *Metabolites*, 3(3):820–837, 2013.
